# 2710. Long Term Use of Contezolid in Lung Transplant Recipients Infected with *Nocardia* Species

**DOI:** 10.1093/ofid/ofad500.2321

**Published:** 2023-11-27

**Authors:** Chunrong Ju

**Affiliations:** The First Affiliated Hospital of Guangzhou Medical University, Guangzhou, Guangdong, China

## Abstract

**Background:**

*Nocardia* commonly infects lung transplant recipients (LTR). Current oral options for long-term therapy may result in intolerance or adverse events. Contezolid is a new oxazolidinone antibiotic with potential less toxicity than linezolid that was approved in China. This study describes the use of contezolid in the treatment of *nocardiosis* among LTR.

**Methods:**

This retrospective study reviewed clinical data of adult LTR who were complicated with *nocardiosis* between January-2018 and December-2022 at the large transplant center China. Diagnosis of *nocardiosis* was confirmed by high resolution CT and cultures from representative samples. *Nocardia spp*. were identified by metagenomic next generation sequencing (mNGS). Drug susceptibility was determined by E-Test for TMP-SMX, Imipenem and Linezolid, and Kirby-Bauer for the others. Patients were followed in hospital and during home therapy for the whole duration of the treatment. Cure was defined by resolution of clinical symptoms, CT scan and culture negative BAL.

**Results:**

During the study period 18 solid organ transplant patients infected with *Nocardia spp.* were identified. Among these patients 14 had pulmonary *nocardiosis* and 4 had disseminated diseases. A total of eight species of *Nocardia* were identified. *Nocardia farcinica* was the most common (5/8); 8 strains of *Nocardia* were resistant to ≥1 common antibiotics (Table 1). Linezolid was the only drug which maintained activity against all *Nocardia spp*. The initial treatment was a combination, usually with TMP-SMX, followed by a continuation regimen reduced to a single antibiotic according to susceptibility. The overall length of combined regimen was 18 days (range 14-25). Linezolid was used in 11 and contezolid in 9 patients, respectively. In 4 patients linezolid was switched to contezolid due to thrombocytopenia (4), anemia (1), and leucopenia (4). The median length of treatment in patients receiving contezolid was 110 days (range 90-210). Overall 16/18 patients were cured, including all patients who received contezolid. Patients’ characteristics, treatment and outcomes are displayed in Table 2.

**Figure:**
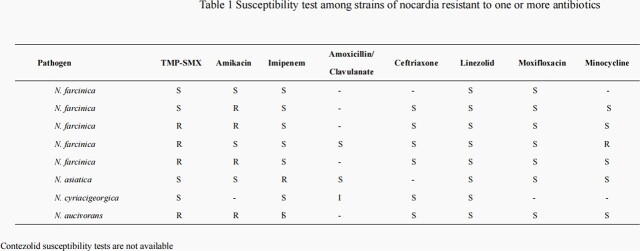

**Figure:**
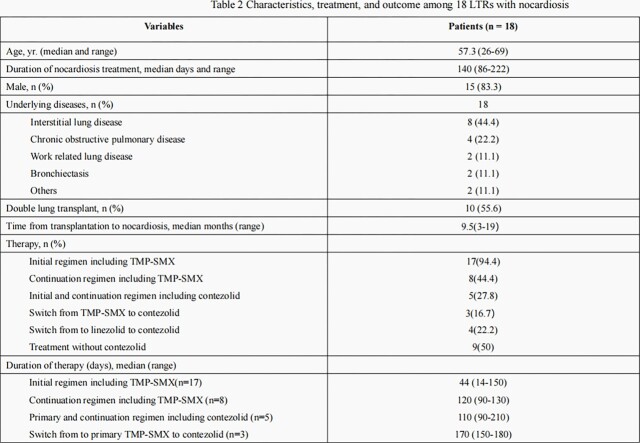

**Conclusion:**

Our study suggests that contezolid can be used in the long-term treatment of *nocardiosis* among lung transplant recipients.

**Disclosures:**

**All Authors**: No reported disclosures

